# Tyrphostins reduce chemotherapy-induced intestinal injury in mice: assessment by a biochemical assay

**DOI:** 10.1038/sj.bjc.6602324

**Published:** 2005-01-18

**Authors:** Y Zlotnik, M Patya, A Vanichkin, A Novogrodsky

**Affiliations:** 1 Felsenstein Medical Research Center, Sackler Faculty of Medicine, Tel Aviv University, Rabin Medical Center, Beilinson Campus, Petah-Tikva 49100, Israel

**Keywords:** tyrphostins, chemotherapy, chemoprotection, intestinal injury, gamma-glutamyl transpeptidase

## Abstract

Intestinal injury that results from chemotherapy belongs to the major factors of dose-limitation in tumour therapy. The tyrphostins AG1714 and AG1801 reduce cisplatin and 5-FU-induced small intestinal mucosal damage, using a quantitative biochemical assay. The assay is based on the determination of the enzymatic activity of gamma-glutamyl transpeptidase, a marker of the brush border epithelium of the small intestine.

Intestinal mucositis is a common complication for a wide variety of anticancer drugs (Khan and Wingard, 2001; Duncan and Grant, 2003).

Clinical trials demonstrated a chemoprotective effect of amifostine (Phan *et al*, 2001), a granulocyte–macrophage colony stimulating factor (Meropol *et al*, 1999) and glutamine (Daniele *et al*, 2001) against chemotherapy-induced intestinal toxicity. We have reported the protective effect of the tyrphostin AG1714 against chemotherapy-induced toxicity without impairing its antitumour efficacy in mice (Novogrodsky *et al*, 1998). Epithelium injury was assessed by histopathological analysis. Here, we present data on the prevention by the tyrphostins AG1714 and AG1801 of cisplatin and 5-fluorouracil (5-FU)-induced intestinal mucosal damage, using a quantitative biochemical assay. The assay is based on the determination of the enzymatic activity of gamma-glutamyl transpeptidase (GGT), a marker of the brush border epithelium of the small intestine (Tate and Meister, 1981; Ferraris *et al*, 1992).

## MATERIALS AND METHODS

### Mice

CD1 female mice (20–25 g) were obtained from Harlan, Jerusalem.

The experimental protocol was approved by the Committee for Care and Use of Laboratory Animals, Rabin Medical Center-Beilinson Campus.

### Materials

AG1714 and AG1801 were synthesised as described (Gazit *et al*, 1989). Cisplatin, (Sigma, Israel) was dissolved in DMSO (Burdick & Jackson Division, Baxter Healthcare, Muskegon, MI, USA) to obtain a solution of 50 mg ml^−1^ and was diluted in saline to 1.0 mg ml^−1^. 5-Fluorouracil (5-FU), 50 mg ml^−1^, was obtained from ABIC, Israel and diluted in saline. Doxorubicin, 2.0 mg ml^−1^, was obtained from TEVA, Israel, and diluted in PBS.

### Formulations

For intraperitoneal (i.p.) injections, tyrphostins were dissolved in cremophor (Sigma, Israel)–absolute ethanol (1 : 1 w/w^−1^) and diluted in PBS (without calcium and magnesium). For oral administration, tyrphostins were dissolved in polyethylene glycol (PEG 400) (Sigma, Israel).

### Treatment protocol

Tyrphostins' solutions were freshly prepared immediately before use. They were injected i.p. in a volume of 0.2–0.4 ml 2 h prior to the cytotoxic agents, or administered orally in a volume 0.2 ml, 4 h prior to the administration of the cytotoxic drugs. Control mice were administered with vehicle solutions.

### Gamma-glutamyl transpeptidase assay

Mice were killed by cervical dislocation.

A segment of the jejunum (approximately 5 cm) was isolated, cut and placed into a tube containing ice cold PBS (2.5 ml). The intestinal segments were then flushed with 10 ml of PBS. In total, 2 × 2 cm^2^ segments were (for duplication) cut, and placed into 1.0 ml of 1.0% Triton X-100, 0.15 M NaCl, 100 mM Tris, pH 8.0 (Tris-Triton buffer) and stirred for 2 min. After 30 min in ice, the tubes were centrifuged and the supernatants were diluted 1 : 1 with Tris-Triton buffer. A measure of 0.02 ml were added to a reaction mixture containing: 0.3 ml of 100 mM glycyl-glycine, pH 8.0, 0.08 ml Tris-Triton buffer, 0.5 ml of 5 mM gamma-glutamyl-*p*-nitroanilide (dissolved in 100 mM Tris, pH 8.0) (final volume, 0.9 ml). All the reagents for GGT determination were obtained from Sigma, Israel.

After incubation for 10 min in a shaking water bath at 37°C, the reaction was stopped by placing the tubes in ice and adding 0.1 ml of glacial acetic acid. After centrifugation, supernatants were read at 405 nm (ELISA). Results were expressed as units of GGT activity per cm of jejunum. One unit is defined as GGT activity, which releases 1.0 *μ*mol of *p*-nitroaniline in 1 h.

### Statistical analysis

The results were expressed as means±s.e.m. Differences among the treatment groups were evaluated using the two-tailed Student's *t*-test. *P*-values of <0.05 were considered statistically significant.

## RESULTS

**Figure 1:**
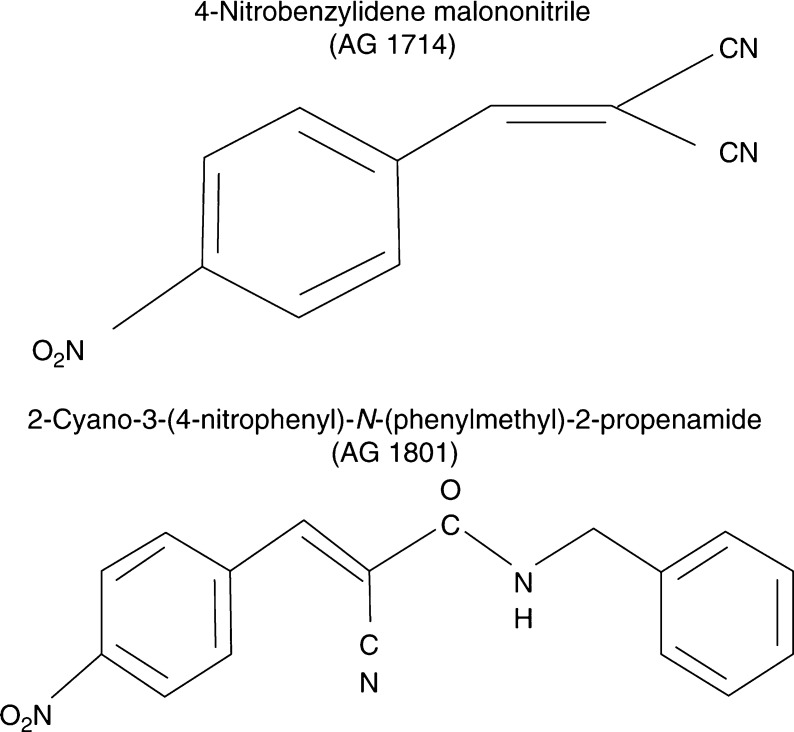
Structure of AG1714 and AG1801.

Figure 1 illustrates the chemical structure of AG1714 and AG1801.

**Figure 2:**
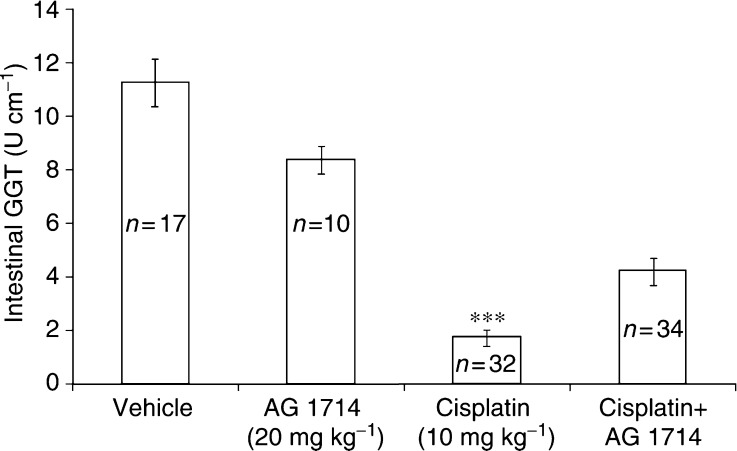
Effect of AG1714 (i.p.) on cisplatin-induced reduction of intestinal GGT. CD1 mice were injected (i.p.) with AG1714, 2 h prior to injection (i.p.) of cisplatin. Mice were killed 4 days after cisplatin administration and small intestinal GGT was determined as described in Materials and Methods. The number of mice (*n*) in each experimental group is depicted in the figure. Figure includes data from five different experiments. Results are expressed as units of GGT activity per 1 cm of small intestinal jejunum±s.e.m. ^***^P<0.001, mice treated with cisplatin+AG1714 *vs* cisplatin alone.

As depicted in Figure 2, cisplatin (10 mg kg^−1^), injected intraperitoneally (i.p.), markedly reduced the content of the GGT in the small intestine jejunum mucosa of mice, as determined 4 days later. Administration of AG1714 (20 mg kg^−1^ i.p.) 2 h prior to injection of cisplatin, abrogated the cisplatin effect by more than two-fold. AG1801 is a structural analogue of AG1714 that shares with it chemoprotective properties such as reduction of chemotherapy-induced mortality and myelosuppression. AG1801 is effective at a lower dose compared to AG1714. Maximal chemoprotective effect of the tyrphostins, administered orally, was attained at higher doses compared to i.p. administration (unpublished observations).

AG1801 is also effective in attenuating chemotherapy-induced small intestinal injury as assessed by determination of GGT activity in the jejunum mucosa.

**Figure 3:**
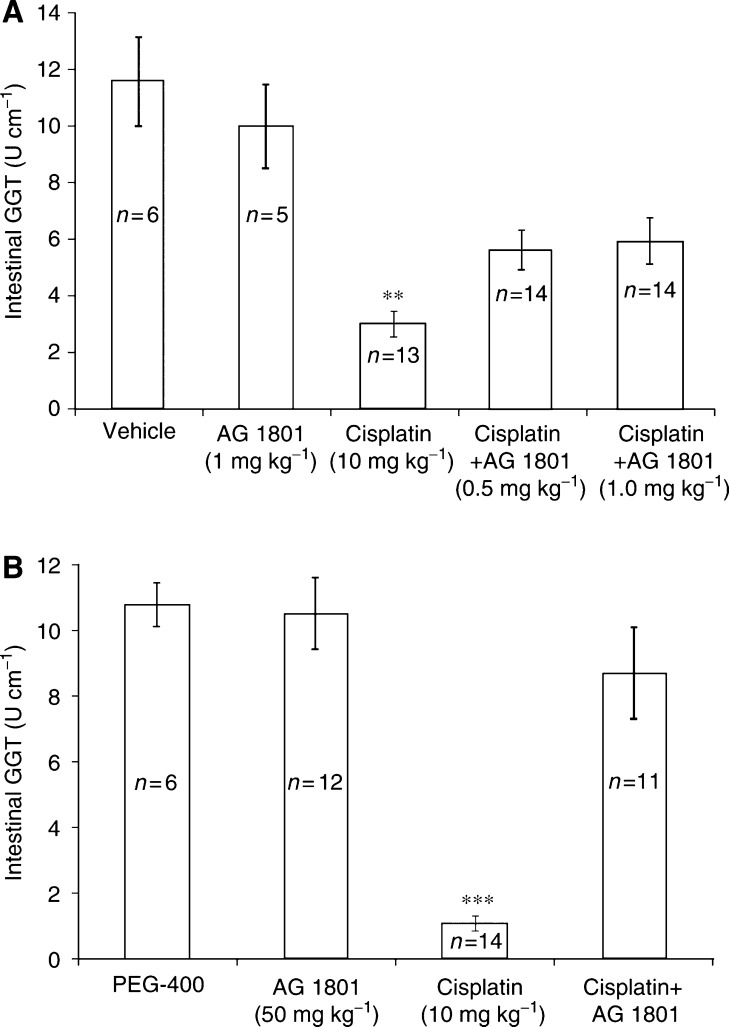
(**A**) Effect of AG1801 (i.p.) on cisplatin-induced reduction of intestinal GGT. Experimental conditions were similar to those outlined for Figure 1, except that AG1801 (at the doses indicated, i.p.) was used. Figure includes data from three different experiments. ^**^P<0.01, mice treated with cisplatin+AG1801 *vs* cisplatin alone. (**B**) Effect of AG1801, administered orally, on cisplatin-induced reduction of intestinal GGT. Mice were injected (i.p.) with cisplatin (10 mg kg^−1^), 4 h after oral administration (by an intragastric tube) of AG1801 (50 mg kg^−1^). Intestinal GGT was determined 4 days after cisplatin administration. Figure includes data from three different experiments. ^***^P<0.001, mice treated with cisplatin+AG1801 *vs* cisplatin alone.

As depicted in Figure 3A, AG1801 (0.5–1.0 mg kg^−1^), administered intraperitoneally 2 h prior to cisplatin, abrogated the cisplatin (10 mg kg^−1^, i.p.) effect by almost two-fold. Oral administration of AG1801 (50 mg kg^−1^), 4 h prior to cisplatin, almost completely prevented cisplatin (10 mg kg^−1^, i.p.)-induced small intestinal injury (Figure 3B).

**Figure 4:**
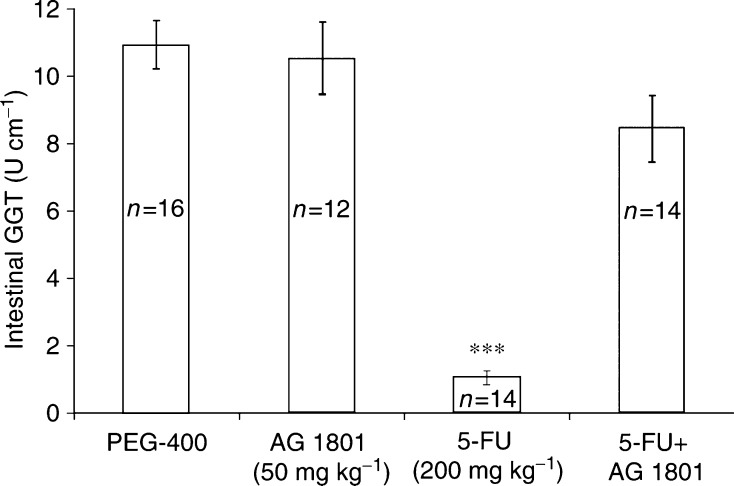
Effect of AG1801 (administered orally) on 5-FU-induced reduction of intestinal GGT. Mice were injected (i.p.) with 5-FU (200 mg kg^−1^) 4 h after oral administration of AG1801 (50 mg kg^−1^). Intestinal GGT was determined 4 days after cisplatin administration. Figure includes data from three different experiments. ^***^P<0.001, mice treated with 5-FU+AG1801 *vs* 5-FU alone.

AG1801, administered orally was also effective in reducing 5-FU-induced small intestinal injury. Oral administration of AG1801, 4 h prior to 5-FU, markedly reduced 5-FU (200 mg kg^−1^, i.p.)-induced small intestinal injury (Figure 4).

## DISCUSSION

Using the quantitative biochemical GGT method, we demonstrated the protective effect of the tyrphostins AG1714 and AG1801 against chemotherapy-induced small intestinal injury. We have previously demonstrated the protective effect of AG1714 against cisplatin induced small intestinal toxicity, using histological analysis (Novogrodsky *et al*, 1998). The biochemical method supplements histological analysis, and has the advantage of providing an objective quantitative assessment of the integrity of the mucosa in a large segment of the intestine.

Oral administration of the tyrphostins seems to be more effective than the intraperitoneal route. This may be due to the direct access of the tyrphostins to the intestinal mucosa.

Chemotherapeutic agents induce intestinal toxicity by an apoptosis-mediated mechanism (Potten *et al*, 1997; Papaconstantinou *et al*, 2001).

We have previously (Novogrodsky *et al*, 1998; Vanichkin *et al*, 2002) postulated that the chemoprotective effect of the tyrphostins is attributed to their ability to selectively inhibit the induction of apoptosis in normal cells but not in cancer cells.

The molecular targets of AG1714 and AG1801 are unknown. These compounds are structurally related to the tyrphostin family that selectively inhibits protein tyrosine kinases (Levitzki and Gazit, 1995). Inhibitors of tyrosine kinases were shown to modulate apoptosis induced by a variety of agents in different cell types (Uckun *et al*, 1992; Bergamaschi *et al*, 1993; Liu *et al*, 1994; Ji *et al*, 1995). It should be noted, however, that AG1714 and AG1801 are not derivatives of hydroxylbenzylidene malononitrile like most of the reported tyrosine kinase inhibitors of the tyrphostin family. Thus, it is not at all certain that the effect of AG1714 and AG1801 reported here is related to the inhibition of protein tyrosine kinases(s). Moreover, as noted above, these compounds are effective upon administration 2–4 h prior to chemotherapy. The i.p. (10 mg kg^−1^) bioavailability of AG1801 was calculated to be about 26% and the oral (20 mg kg^−1^) bioavailability was calculated to be 21%. However, AG1801 is cleared rapidly from the blood (*T*_1/2_, is less than 10 min). Hence, at the time of the administration of chemotherapy, the blood level of AG1801 may be very low. AG1801 may elicit its effect by providing a signal, rendering the cell resistant to the induction of apoptotic injury. It is also possible that a degradation product of AG1801 rather than the native compound elicits the biological effect.

The study reported here was confined to biochemical monitoring of acute small intestinal injury in the mouse induced by chemotherapy. The GGT method is not suitable for assessment of large intestinal damage due to the low content of GGT. The applicability of this method to other species or other types of intestinal damage, such as those associated with inflammatory processes, requires further investigation. In this context, it should be noted that GGT in different cell types is subject to phenotypic alterations induced by different agents (Novogrodsky *et al*, 1976; Wasserman *et al*, 1987; Sidi *et al*, 1988).

It is very difficult to quantitatively assess the sensitivity of biochemical GGT assay in comparison to the histological assay. However, The intestinal injury induced by chemotherapy is quite often nonhomogenous and therefore the biochemical assay that analyses a long segment of the intestine (centimeters) is superior to the histological assay that analyses a very small segment (microns).
